# Differentiation of Pluripotent Stem Cells Into Thymic Epithelial Cells and Generation of Thymic Organoids: Applications for Therapeutic Strategies Against APECED

**DOI:** 10.3389/fimmu.2022.930963

**Published:** 2022-06-29

**Authors:** Nathan Provin, Matthieu Giraud

**Affiliations:** Nantes Université, INSERM, Center for Research in Transplantation and Translational Immunology, UMR 1064, Nantes, France

**Keywords:** thymus, IPSC, thymic epithelial cells (TEC), differentiation, APECED, tolerance

## Abstract

The thymus is a primary lymphoid organ essential for the induction of central immune tolerance. Maturing T cells undergo several steps of expansion and selection mediated by thymic epithelial cells (TECs). In APECED and other congenital pathologies, a deficiency in genes that regulate TEC development or their ability to select non auto-reactive thymocytes results in a defective immune balance, and consequently in a general autoimmune syndrome. Restoration of thymic function is thus crucial for the emergence of curative treatments. The last decade has seen remarkable progress in both gene editing and pluripotent stem cell differentiation, with the emergence of CRISPR-based gene correction, the trivialization of reprogramming of somatic cells to induced pluripotent stem cells (iPSc) and their subsequent differentiation into multiple cellular fates. The combination of these two approaches has paved the way to the generation of genetically corrected thymic organoids and their use to control thymic genetic pathologies affecting self-tolerance. Here we review the recent advances in differentiation of iPSc into TECs and the ability of the latter to support a proper and efficient maturation of thymocytes into functional and non-autoreactive T cells. A special focus is given on thymus organogenesis and pathway modulation during iPSc differentiation, on the impact of the 2/3D structure on the generated TECs, and on perspectives for therapeutic strategies in APECED based on patient-derived iPSc corrected for *AIRE* gene mutations.

## Introduction

Immune tolerance is primarily set in the thymus with the generation of non-autoreactive T cells originated from the maturation of thymocytes through intimate interactions with specialized sets of thymic epithelial cells (TECs). These complex interactions enable the selection of maturing thymocytes for the functionality and non autoreactivity of their T cell receptors (TCR). The step of negative selection, which is responsible for the induction of thymocyte self-tolerance, is mediated by TECs that reside in the thymic medulla and express the autoimmune regulator (AIRE) (1). Loss-of-function mutations in the *AIRE* gene result in the rare autoimmune polyendocrinopathy candidiasis ectodermal dystrophy syndrome (APECED), a life-threatening autoimmune disease characterized by severe autoimmune lesions in peripheral tissues (2). The direct cause of this syndrome is the impairment of negative selection due to AIRE deficiency, resulting in the escape of autoreactive T cells into the periphery. Current treatments of APECED are only symptomatic including the administration of immunosuppressants, antifungal drugs with a constant monitoring of candidiasis infection, and hormone replacement. Although these therapeutics have improved the course of APECED, they don’t address the root cause of the disease, leaving the patients at risk of premature death (3). Induced pluripotent stem cells (iPSc) are a promising tool for the development of new cellular and genetic therapies for APECED. These cells are reprogrammed somatic cells obtained by the induced expression of four genes (*OCT4*/*POU5F1*, *KLF4*, *SOX2*, *MYC*) (4). The remarkable proliferation potential of iPSc and their ability to differentiate into various cell fates (5–7) have paved the way to promising therapeutic strategies aiming to restore tissue functions, notably in rare genetic diseases. Several iPSc-derived cell therapies against various pathologies such as cancer, autoimmune disorders or Parkinson’s disease are currently being evaluated by clinical trials (8–10). However, some concerns have been raised regarding the overall safety of stem-cell based therapies, since reprogramming of somatic cells can lead to enhanced mutation susceptibility and selection of deleterious mutations originally present in a minority of somatic cells. In addition, with the implication of *OCT4* in tumorigenesis, the risk of inducing teratoma and malignant tumor formation is to be seriously considered (11). Thus, stringent quality controls of the generated iPSc and their differentiated products, as well as the fine purification of the populations of interest are crucial (12). This review covers the recent developments of iPSc differentiation into TECs, the different approaches used to mimic thymic embryological development and how it can provide new strategies for therapeutic application against APECED.

## Thymus Function: T Cell Maturation and Selection

**Figure 1:**
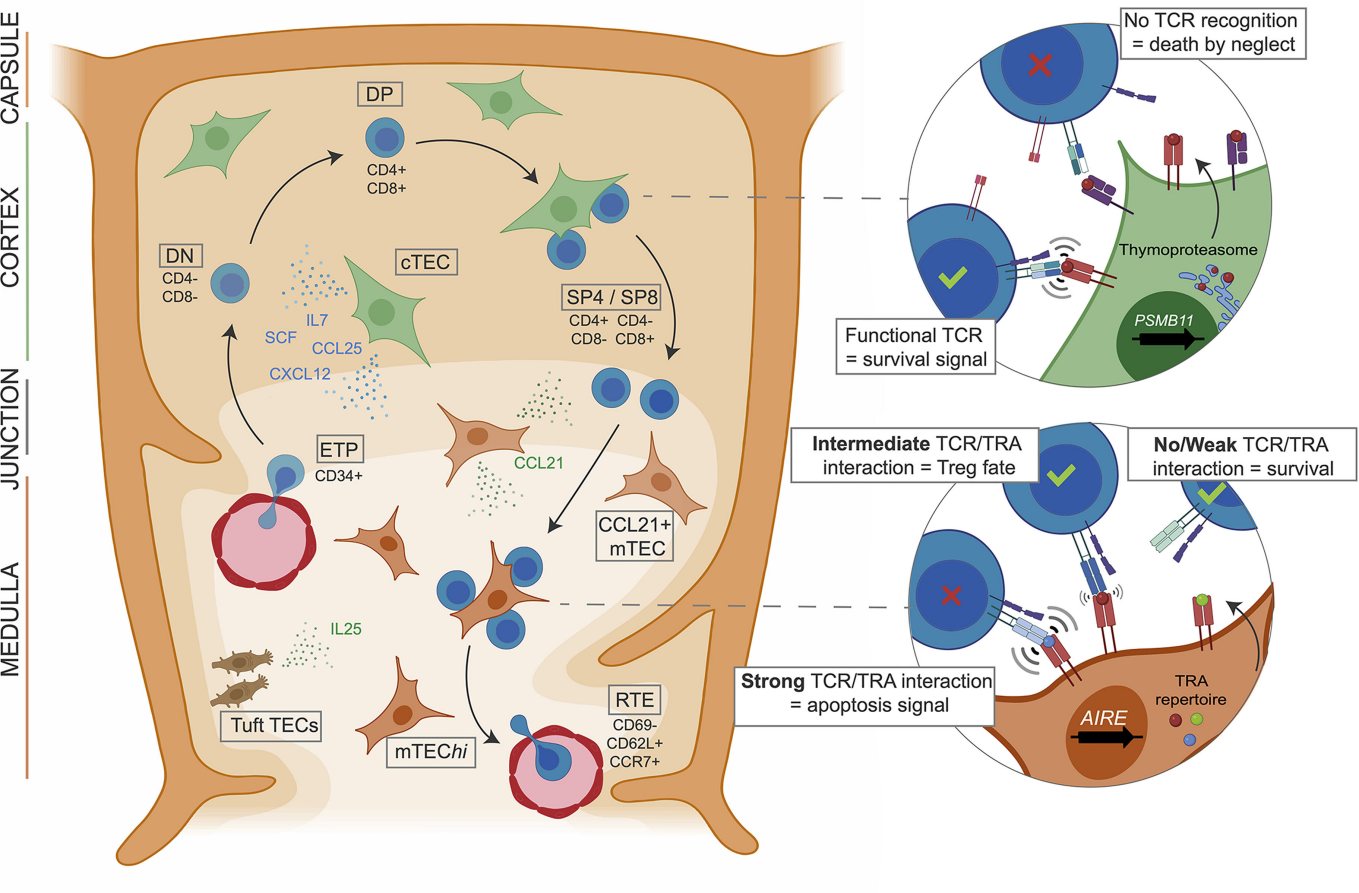
cTEC and mTEC orchestrate thymocyte maturation and selection. Early thymic progenitors (ETP) originating from the bone marrow enter the thymus and are attracted to the cortical region by chimiokines expressed by cTECs, also inducing growth and T lineage commitment by contact with NOTCH ligands. After TCR rearrangement CD4/CD8 DP thymocytes enter positive selection of the functional TCRs mediated by cTEC expressing the thymoproteasome. Resulting SP T cells are attracted to the medulla where thymocytes with auto-reactive TCRs are negatively selected by mature mTECs presenting the self-antigen repertoire under AIRE regulation. Thus, T cells with functional TCRs, able to recognize foreign antigens but tolerant to the self, are generated by the thymus.

In the thymus, maturing T cells undergo several steps of expansion and selection mediated by TECs that account for 0.5% of the thymic cellularity in the adult thymus (13) (**Figure 1**). TECs form the backbone of the stromal compartment and interact with a large number of thymocytes (14). They have been historically separated into cortical TECs (cTECs) and medullary TECs (mTECs) located in the outermost and the core areas of the thymic lobules, respectively. These two populations exhibit different phenotypes and play distinct roles in the control of T cell maturation. cTECs drive the commitment of the early thymic progenitors (ETPs) to the T cell lineage by providing Notch ligands such as Dll4 (15). They also control ETP homing and expansion through the secretion of chemokines and growth factors such as Ccl25, Cxcl12, Scf and Il-7 (16). At the later double-positive (DP) stage, thymocytes with TCR that are able to recognize peptide:major histocompatibility complex (MHC) receive survival signals from cTECs and are thereby positively selected (**Figure 1**). The peptides presented by cTECs are generated by a unique proteasome comprising the cortical marker ß5t encoded by the *Psmb11* gene (15, 17). After the positive selection, thymocytes undergo a step of negative selection aiming to deplete those with a TCR having a high avidity for self-antigen peptides. This step is mediated by mTECs that attract single-positive (SP) thymocytes through the secretion of chemokines like Ccl21 (18). The negative selection is enabled by the unique ability of mTECs to express 90% of the genome (19–21), including the expression of a broad repertoire of tissue-restricted antigen (TRA) genes under the regulation of transcriptional activators such as FEZF2, CHD4 and especially AIRE (22–24). The non-conventional activation factor Aire controls the expression of a large fraction of these TRAs (25, 26) and is specifically expressed in the mature subpopulation of mTECs showing high levels of the major histocompatibility class II (MHCII) molecule (mTEChi) and a high turnover. Importantly, the last decade has seen many aspects of the Aire-driven regulation of TRA expression being uncovered (26, 27). In mice, the expression of TRA genes follows a stochastic order: they are co-expressed in modules randomly present in individual mTEChi spread out in the thymic medulla. This pattern of expression enables a complete screening of the TRA repertoire by the thymocytes passing through the medulla. The intensity of the interactions between TRA peptide:MHCII complexes at the surface of mTEChi and the TCR is determinant for the fate of thymocytes (1). Thus, thymocytes with a strong self-reactive TCR will undergo apoptosis, while those harboring a self-reactive TCR with an intermediate strength will be directed into the natural T regulator (nTreg) lineage which plays a major role in peripheral immune tolerance (28) (**Figure 1**). At their final stage of maturation, the thymocytes will enter the periphery through the cortico-medullary junction vasculature, as recent thymic emigrants (RTEs) characterized by their expression levels of CD69, CD62L (*SELL*), Qa2 and CCR7 (29, 30) (**Figure 1**).

## TEC Heterogeneity and Maturation

From the homing of hematopoietic progenitors to the thymus to the escape of RTEs into the periphery, the maturation of T cells mostly relies on intimate interactions between thymocytes and TECs. These interactions are crucial for the maturation of TECs through a process referred to as thymic crosstalk (31–34). Indeed, TECs need to receive signals such as those mediated by Rankl and Cd40l to mature and survive (35). In recent years, advances in high-throughput single-cell (sc)RNA sequencing (scRNA-seq) have pushed further our understanding of TEC heterogeneity beyond the typical dichotomy between cTEC and mTEC in mice (16, 21, 36) and humans (37–39). Hence, new TEC populations were identified, notably populations composed of atypical tuft cells sharing similarities with intestinal tuft cells (16), myoid-like epithelial cells or neuroendocrine epithelial cells (37). While these populations are well characterized at the transcriptomic level, their functional role in the thymic microenvironment and their potential effect on thymocyte development remain elusive.

scRNA-seq studies of TEC heterogeneity in individuals of different ages also contributed to reveal the existence and identity of a TEC progenitor (TEP) population (36, 38). TEPs exhibit a cortical phenotype characterized by the expression of CD205 and ß5T (encoded by the *LY75* and *PSMB11* genes, respectively) (40–42). They were shown to be the source of mTECs and cTECs in fetal and neonatal thymuses (14, 43, 44). However, there is still a lack of evidence supporting this model in adults. After birth the thymus undergoes involution with a drastic decrease of its activity and cellularity, and shows a shift of the relative cTEC vs mTEC abundance in favor of the mTEC compartment (45, 46). In addition, it is assumed that TEPs enter quiescence in response to BMP4 and Activin A inhibitor follistatin (FSH) signaling (37, 47, 48). Thus, the emerging model for the origin of TECs relies on bipotent cTEC-like fetal TEPs entering quiescence upon aging and giving rise to lineage-restricted immature populations for the replenishment of medullary and cortical TEC compartments. However, additional studies based on single-cell fate-mapping need to be carried out to precisely understand the relationship between TECs and their progenitors upon aging. Another question left unanswered is the nature of signals underlying the medullary or cortical orientation of bipotent TEPs.

Promising results have been obtained over the last years, notably describing the role of Notch modulation in this fate decision (37, 49, 50). Indeed, Notch signaling has been shown to be essential for the mTEC specification of TEPs, notably through its fine-tuned regulation by the chromatin regulator HDAC3 (51). Thus, these findings support a model of a cTEC-like bipotent TEP population undergoing a default cortical differentiation, with Notch signalization promoting the mTEC transcriptional program. Despite the main paradigm of a TEC compartment of exclusive endoderm origin, Chakrabarti et al. recently showed that a population of bone-marrow hematopoietic progenitors transdifferentiate in true *Foxn1*-expressing TECs in the thymus (52). These hematopoietic bone marrow progenitors would migrate in the same manner as Cd34+ thymus seeding progenitors but would further differentiate into epithelial cells expressing cytokeratins and the master regulator of TEC development Foxn1. Moreover, Cd45+ Epcam+ cell could also give rise to Fsp1-expressing thymic fibroblasts. Thus, this fate mapping study identify a bone-marrow originating population able to transdifferentiate into TECs and fibroblasts to replenish the thymic stroma, suggesting that the TEC lineage development is more plastic than previously thought and may involve various progenitor populations originating from different embryological layers. However, proper characterization studies using high-throughput omics are still needed to precisely describe this newly identified cell population.

We mainly focus here on the role of TECs in thymocyte selection given the origin of APECED. However, other cell populations in the thymus have been shown to participate to this process. Indeed, dendritic cells (DC) have also the ability to induce clonal deletion and Treg generation (53–55). Different processes involving multiple DC subpopulations have been described. Briefly, migrating DCs can transport peripheral antigens to the thymus, negatively select thymocytes and induce Tregs (56, 57). Transendothelial DCs located in thymic vasculature capture blood circulating antigens and use them for selection (58, 59). Another source of antigens for DCs directly come from the thymic stroma through a mechanism of intercellular antigen transfer from the TECs (60, 61). Lymphotoxin β receptor (LTβR) expressed by mTECs has been shown to be central in this interaction by controlling the frequency and composition of intrathymic DC populations (62). DCs could also process self-antigens produced by thymic fibroblasts as a complementary source of self-antigens (63). Often neglected, thymic fibroblasts are now revealed as a crucial actor of the thymic microenvironment, with distinct subpopulations involved in functions as diverse as self-antigen production and regulation of both TEC and T cell maturation (63–65). Finally, these alternative sources of autoantigens or presenting cells are supported by studies in which disrupted thymuses with low TEC cellularity and morphological anomalies have no effect on mature T cell population frequency nor on the repertoire diversity (66).

## Thymus Organogenesis

**Figure 2:**
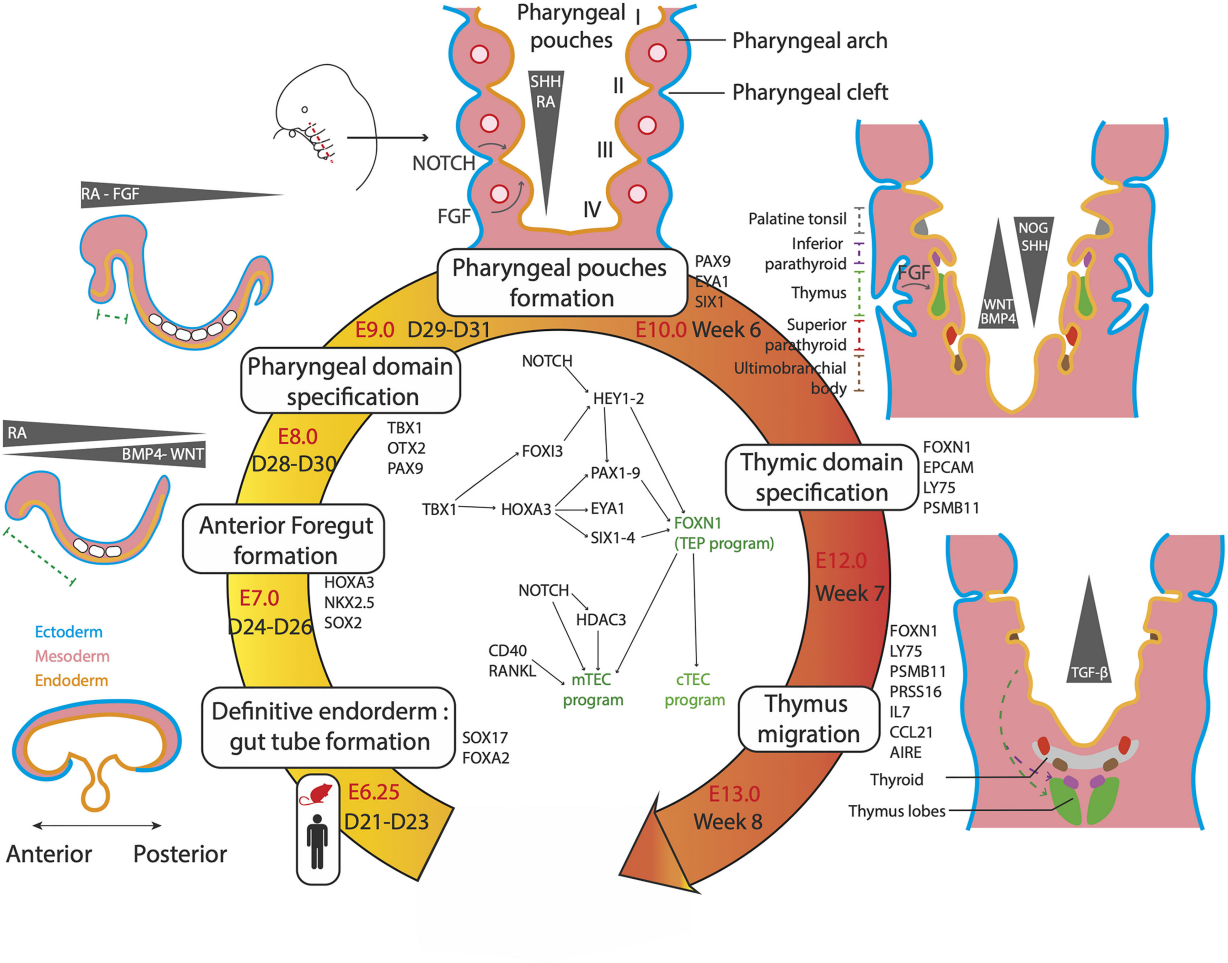
Genetic and molecular networks regulating the thymus organogenesis. The thymus is a definitive endoderm derivative. The main steps of organogenesis of the pharyngeal apparatus and thymus formation are described, with time scales in mouse and human, key markers genes and signalling molecules. First, gradients of cytokines and small molecules pattern the endoderm, resulting in an anterior domain between D24-D26 in human. Further patterning results in formation of the pharyngeal domain and its segmentation in pharyngeal arches at 6 weeks. The 3PP forms under singling involving retinoic acid (RA), Hedgehog (SHH), NOTCH and fibroblast growth factors (FGF). 3PP gives rise to the thymic primordium expressing FOXN1 by week 7. By week 8, the thymic primordium migrates under TGFβ signaling to its final position and TEC mature in cTEC and mTEC.

The first advances in the understanding of thymus organogenesis relied on comparative anatomical observations and histology studies of fetal tissues. These approaches revealed that the thymus is derived from the pharyngeal pouches that are transitory embryonic structures appearing between the third and fourth week of development in humans (67) and from E8 in mice (68, 69) (**Figure 2**). The pharyngeal pouches are invaginations originating from the most anterior foregut endoderm (67, 70, 71). It was shown in human fetuses that the thymus mostly derived from the third pharyngeal pouch (3PP) (72, 73). However, the embryological layer of origin of the thymus has long (74, 75) been uncertain. In the early 2000s, the single endodermal origin was demonstrated after ectopic transplantation experiments of pharyngeal endoderm proving that it is sufficient to give rise to a fully formed and functional thymus (68, 76).

Although thymus organogenesis has been well described in mice (74, 75) the precise cellular and molecular mechanisms governing human thymus development are still elusive. In humans, the thymus forms from the 3PP endoderm at week 7 of development and initiates migration at 8.5 week (77). Involution of the 3PP endoderm results in a stratified epithelium of Cldn3/4-expressing cells polarized around a central lumen (78, 79) showing an early morphogenesis similar to other organs with epithelia organized in branching ducts, such as lung or pancreas. However, the definitive histological structure of the thymus is radically different, with formation of a 3D network of TECs that is far from the stratified epithelium constituting a branching architecture. This key aspect of thymic functionality, allowing the maximization of contacts between thymocytes and TECs, is mainly explained by the expression of the main TEC marker, Foxn1. This gene was first identified as the nude gene, originally described in the eponym hairless mice mutants exhibiting an absence of functional thymus (80). In mice, *Foxn1* expression is detected in the 3PP endoderm as early as E9.5 and reaches high levels at E11 (80, 81). However, *Foxn1* is not directly responsible for the commitment to thymic epithelial cell fate, since ectopically transplanted E9 3PP tissues, which do not express *Foxn1* yet, are still able to generate fully developed thymuses (68). Nude mice show normal thymic primordium formation and migration but impaired maturation of the TEC compartment and consequently of T cell colonization (82). Thus, *Foxn1* may be downstream of a regulatory network driving the commitment to thymic epithelial progenitors but would play a central role in the differentiation of TEPs into TECs (44, 68, 80, 83). Thymus rudiments from *Foxn1* deficient mice shows an atypical branching structure, with formation of multiple lumens giving rise to a fully develop ductal system similar to the pancreas. In addition, ectopic expression of Foxn1 results in impaired epithelium formation and absence of lumen (84), showing Foxn1 ability to disrupt the classical tubular morphogenesis program. Finally, Foxn1 has been shown to be necessary for the expression of a full set of factors that control TEC transcriptional programs, such as *Cxcl12*, *Ccl25*, *Dll4* and MHCII genes (85). More recently, *Foxn1* has also been shown to control the expression of *Cd40*, *Cd80*, *Aire* and *FgfrII* that are crucial for TEC differentiation, amplification and function (85–87). Thus, by inhibiting tubulogenesis of the thymic epithelium and inducing expression of key genes of the TEC program, Foxn1 allows the structuration of the thymic environment and TEC generation (88).

### Molecular Regulatory Networks in 3PP and Thymus Organogenesis

A complex interplay between the neural crest cells, the mesoderm-derived mesenchyme and the 3PP endoderm controls the fate, migration and expansion of cell populations in the developing thymus. The main genetic regulatory network is composed of the *TBX1*-*HOXA3*-*PAX9* and *EYA1*-*SIX1* cascades that are regulated by a set of signaling molecules secreted by the neural crest and mesoderm cells, such as retinoic acid (RA), proteins of the Wingless-int (WNT) family, bone morphogenetic proteins (BMP), fibroblast growth factors (FGF) and sonic hedgehog (SHH) proteins (**Figure 2**).

These factors, which are secreted by the mesoderm core and the neural crests in addition to the endoderm, guide the development of the thymic primordium. RA is a small non-peptidic vitamin A derivative that plays a key role throughout the embryonic development (89, 90). Gradients of RA have been shown to regulate the posterior pouch formation in several species (91–93). Disrupting RA activity during the development results in the absence of formation of posterior pharyngeal pouches (91, 93, 94). Moreover, RA was shown to be a key player in the early formation of pharyngeal pouches by regulating the expression of genes of central importance in their development, such as *TBX1*, *HOXA3*, *PAX1* and *PAX9* (85, 87, 89, 90, 95) (**Figure 2**). Proteins of the WNT family, including WNT4b and WNT5a (96–98), are expressed in the pharyngeal pouches and lead to the upregulation of *FOXN1* by activating the canonical WNT/beta catenin pathway (96). Thus, modulation of the WNT signaling is critical to the formation of the thymic primordium and the maintenance of the thymic postnatal epithelium (97, 99, 100). Several studies have shown that genes of the WNT family are down-regulated in aged involuted thymuses (101–103) and are expressed in TECs under FOXN1 positive regulation (86, 100). However, a strong WNT signaling is detrimental to the thymic development (104) highlighting the importance of a proper modulation of WNT signals in TEC physiology. In mice, modulation of the Bmp pathway through a Bmp4-Noggin gradient in the thymic and parathyroid primordia, is responsible for a correct organ separation and thymic capsule formation (105, 106). Bmp4 has been shown to directly upregulate *Foxn1* and *FgfrIII* (107). BMP signaling is thus closely integrated into FGF pathways that have been shown to play a crucial role in 3PP and thymic development (108, 109). Indeed, mutant mice for *Fgf8* (110) and *Fgfr2-IIIb*, a receptor of Fgf7 and Fgf10 (87) show an impaired thymus development and arrest of TEC maturation. In zebrafish, the secretion of FGF8 in closeby mesoderm directs 3PP formation. However, later FGF signal inhibition through Spry is also necessary for thymic primordium migration and TEC proliferation (111). Similarly to the above molecules, Hedgehog plays multiple regulatory roles in the thymus. During the later steps of TEC maturation, SHH negatively impacts TEC proliferation but stimulates MHCII expression (112). SHH expression is restricted to the anteriormost pharyngeal apparatus, both in the endoderm and mesoderm, and plays a role in pharyngeal pouch patterning (113). At E10.5 SHH endorses a dorsalizing role, contrasting with the ventral thymic fate instructed by BMP4. These clues, added to the fact that SHH endodermal expression represses *FOXN1*, indicate that SHH signal inhibition is necessary to promote a thymic over parathyroid fate (114, 115). Overall, all these factors are involved in an integrated regulatory network orchestrating the specification, maturation and migration of the pharyngeal pouch derivatives.

### Axis Patterning of the Definitive Endoderm and Emergence of the Foregut

Since the 3PP is a structure of the pharyngeal domain that is the anterior-most segment of the foregut endoderm, the identification of the molecular signals that sustain definitive endoderm patterning is a prerequisite for the control of the first steps of iPSc differentiation towards TECs. In mice, definitive endoderm specification is initiated during the gastrulation at E6.25, after which morphogenetic processes occur leading to the formation of the tubular gut structure by E8.0 (116) (**Figure 2**). Most of the pathways involved in pharyngeal pouch formation have also been shown to orchestrate the antero-posterior patterning of the gut tube. Indeed, it is now well established that the Bmp-Wnt-Fgf signaling has a posteriorizing effect on endoderm development and that its inhibition is required for the acquisition of foregut identity (117–119). In addition, the TGFß/Nodal inhibition and the RA signaling are also involved in the anteriorization of the endoderm (117). Recently, scRNA-seq datasets of foregut endoderm have been generated in mice between E3.5 and E12.5 (116, 120–122), including foregut mesoderm (116) and pharyngeal endoderm (122) lineages, thereby constituting atlases of endodermal and mesodermal cell populations involved in pharyngeal development. Hence, sets of genes specific to different stages of pharyngeal development could be identified, such as *Pax9* and the *Eya1*-*Six1* cascade for pharyngeal endoderm at E9.5 (**Figure 2**). More generally, these studies provide a model of pharyngeal endoderm development of unprecedented precision. In the case of the thymus, an early ventral foregut endoderm population expressing *Nkx2-3* and 2-5 at E8.5 gives rise to the ventral pharynx expressing *Bmp4* at E9.5 (116). It appeared that the key pathways involved at this stage of differentiation include FGF, NOTCH and RA signals arising from the surrounding mesoderm, as well as BMP autocrine ligands. Among the previously unrecognized pathways involved in anterior foregut development are those driven by HIPPO (121), EGF and NGF (120). At E10.5, the 3PP undergoes its formation upon activation of *Eya1* and *Six1*. This cell population further differentiates into progenitors of parathyroid, ultimobranchial bodies and TEPs that begin to express *Foxn1* at E11.5 and eventually give rise to early cTEC and mTEC populations at E12.5 (122).

Comfortingly, the same populations and signaling pathways are found in comparative studies with human embryos, validating that foregut endoderm organogenesis is conserved between mice and humans (77, 120). Finally, these studies provide key insights into which signaling pathways need to be modulated to direct differentiation of pluripotent human cells into TECs, even if most of the results presented above need to be confirmed by proper *in vivo* knock-out or lineage tracing studies.

## Differentiation of Pluripotent Stem Cells Into the Thymic Lineage

**Figure 3:**
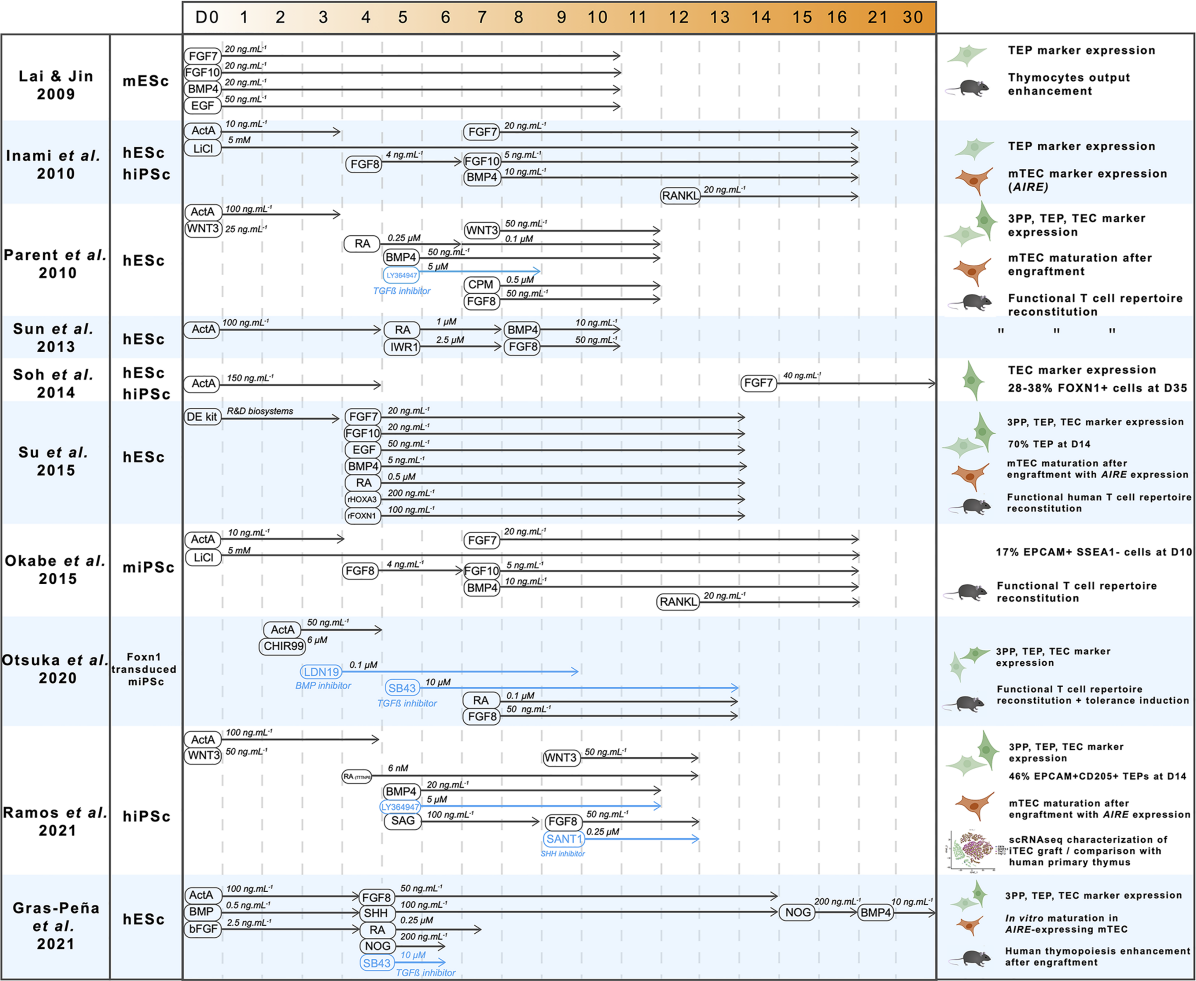
A synthesis of pluripotent stem cells thymic differentiation strategies. A decade of advances in thymic differentiation resulted in perfected protocols allowing production of mature mTEC by mimicking the thymic organogenesis *in vitro*. For each reference, cell type used, pathway-modulating molecules timings and concentrations are indicated, as well as results in terms of nature of the obtained cells and their ability to induce T cell maturation. ActivinA (ActA), Fibroblast growth factors 7,8,10 (FGF7-8-10), Bone Morphogenetic Protein 4 (BMP4), LiCl (Lithium Chloride), Retinoic acid (RA), Cyclopamine (CPM, hedgehog inhibitor), Sonic Hedgehog (SHH), LY364947 (TGFβ inhibitor), SB43 (TGFβ inhibitor), Wingless family member 3 (WNT3), IWR1 (WNT inhibitor), Epidermal Growth Factor (EGF), CHIR99 (WNT3 agonist), LDN19 (BMP inhibitor), Noggin (NOG), SANT1 (Hedgehog inhibitor), Smoothened Agonist (SAG, Hedgehog agonist).

Thanks to the growing understanding of signals driving the formation of the thymus, considerable progress has been made in the differentiation of pluripotent stem cells into the thymic epithelial lineage. We hereby review the different approaches, their achievements and limitations. A pioneer work was carried out by Lai & Jin, who successfully differentiated mouse embryonic stem cells (mESc) into cells showing a TEP phenotype (123) (**Figure 3**). Using a combination of FGFs, EGF and BMP4, they obtained a 24% proportion of EPCAM-positive cells after 10 days in a monolayer culture system. Despite this low proportion, EPCAM+ cells showed the expression of the TEP markers *Pax1*, *Pax9* and *Foxn1*. These induced TEPs were able to successfully reconstitute a cortical and medullary compartment 6 weeks after engraftment under the kidney capsule, confirming their nature of bipotent progenitors. This regenerated thymus contained TCRαß+ CD3+ thymocytes expressing CD4 and CD8 in similar proportions than in native thymus.

Further advances were made by Inami et al. one year later (124). Using human iPSc and by adding RANK ligand (RANKL) to an optimized cytokine cocktail at day 12, they not only reproduce the differentiation into TEP with protein expression of their markers *Pax1*, *Krt5*, and Notch ligands DLL4 and DLL1, but for the first time they detected low levels of *AIRE* expression indicating further maturation of TEPs into mature mTECs. However, the functionality of this mTEC-enriched differentiated population was not addressed in this study, nor its heterogeneity which is an important parameter to control since a significant proportion of unwanted cell lineages are expected to arise from this differentiation and hinder inter-experimental reproducibility. In addition, the poor understanding of late TEC development and the lack of specific markers were major obstacles for a thorough analysis of the differentiated cells. To identify a combination of cell surface markers that are specific to TEPs, Soh et al. designed hESc reporter lines with a GFP cassette inserted into the exon 2 of the *FOXN1* gene locus (125). Surprisingly, the differentiation of these hESc was successfully performed using a simple protocol consisting of exposure to Activin A for the first 4 days and supplementation of FGF7 from day 14 in embryoid bodies (**Figure 3**). Depending on the cell line, this protocol resulted in a strong proportion of GFP-positive cells (27-37%) after 35 days. Consistent with engagement in the thymic epithelial lineage, this GFP+ population was positive for TEC-specific markers such as KRT5, KRT14 and Notch ligands JAG2 and DLL4. However, characterization of the differentiated cells has not been performed further, leaving unanswered their maturation stage as well as their cortical and medullary identity. In addition, these cells were not able to support thymopoiesis since coculture with CD34+CD7+ proT cells failed to result in thymocyte differentiation.

Most of the progress made on the differentiation of pluripotent stem cells into TECs came from the studies of Parent et al. and Sun et al. that sought to optimize the differentiation protocol by monitoring the expression of markers of the intermediate foregut and pharyngeal pouch endoderm stages after cultivating hESc with different combinations of factors (126, 127) (**Figure 3**). These two studies showed that RA is needed for anteriorization of definitive endoderm, as well as BMP4 and WNT3 for acquisition of TEP identity. In addition, inhibition of WNT by IWR1, of TGFß by LY364947 at day 5, and Hedgehog by Cyclopamin (CPM) from day 7 to day 11 was shown to be required to increase FOXN1 expression. However, absence of unbiased experimental design did not allow an unambiguous and accurate identification of the effect of each factor on thymic differentiation, likely resulting in suboptimal results. Both protocols resulted in a significant upregulation of markers of thymic identity. However, no markers of TEC maturation were detectable. The induced TEPs were reaggregated and transplanted into nude mice to maturate further as described by Lai & Jin. The graft matured and was able to support thymopoiesis and reconstitute peripheral blood T cell compartments (127). Spectratype analysis of the TCR repertoire showed increased TCR Vß rearrangement diversity in mice engrafted with hESc-derived TEPs compared with controls (127). CD4+CD25+FOXP3+ Treg were also detected in engrafted mice (127). Importantly, the T cells generated in engrafted nude mice were functional showing IL2 secretion and proliferation after stimulation. They were also able to reject skin grafts. Since this model relies on cross-species reactivity, the same experiments were carried out in humanized mice with human hematopoietic progenitors. Similar results were obtained, therefore confirming the ability of thymic grafts to induce human T cell generation.

Additional approaches based on different strategies than directed differentiation, showed that the key factor FOXN1 was sufficient to induce TEC differentiation. First clues came from the study of Su et al., showing that culturing hESc with recombinant HOXA3 and FOXN1 resulted in significantly increased TEP yield (128). Additional evidence came from the reprogramming of fibroblasts by *FOXN1* over-expression (86) showing that it is sufficient to drive differentiation towards the thymic epithelium fate with large, polygonal cells resembling TECs and expressing factors that sustain thymocyte development such as DLL4 and CCL25. These induced TECs (iTECs) were also able to mature ETPs into CD4+ and CD8+ SP T cells, both *in vitro* after 12 days of coculture and *in vivo* after engraftment in mice. In these mice, T cell functionality was confirmed with increased IL2 secretion in response to CD3/CD28 stimulation. These findings revealed that TECs can be generated from fibroblasts through the sole overexpression of *FOXN1* thus highlighting a key role of this transcription factor in driving the TEC fate program. Together with the advancement of the delineation of the regulatory cascade inducing thymic fate, these results confirm that FOXN1 is necessary and sufficient for the induction of the TEC program, even if it does not induce thymic fate by itself. However, the reliability of the reprogramming approach to reconstitute the TEC compartment with its full heterogeneity needs to be evaluated and compared to the directed differentiation of iPSc that mimics thymus organogenesis. Several other studies have replicated these results, through directed differentiation alone (129) or in conjugation with *Foxn1* overexpression (130, 131) and raised the question of induction of immune tolerance. As expected, *Foxn1* overexpression in mouse iPSc significatively enhances the differentiation into TEPs, resulting in cells that express TEP markers *Pax9*, *Dll4* and *Foxn1* (129, 130) and undergo a proper TEC maturation after engraftment in mice. The effect of these iPSc-derived thymic grafts on immune tolerance has been studied by grafting skin biopsies from the same donor mouse strain from which the iPSc were generated in a recipient of another mouse strain after immune depletion by irradiation and anti-T antibody treatment (130) or directly in nude mice (129). Interestingly, recipient mice with a B6-derived thymic graft showed increased B6 skin graft survival. However, the cellular mechanisms leading to the induction of self-tolerance after iPSc-derived thymic graft needed to be more thoroughly examined.

More recently, comprehensive studies came out and pushed this topic further. Indeed, Ramos et al. and Gras-peña et al. optimized the differentiation protocols, notably by modulating temporal Hedgehog signaling (132, 133) (**Figure 3**). Interestingly, both protocols include Hedgehog activation during the step of pharyngeal endoderm induction, contrary to what was done in a previous report (126). Hence, the fine temporal modulation of the Hedgehog-specific pathways may be key to trigger a proper differentiation towards pharyngeal endoderm. Other examples of temporal modulations are also observed with the inhibition of BMP through Noggin between day 15 to day 21 followed by a more classical activation of BMP4 from day 21, resulting in a significant 10-fold increase of *PAX9* expression (133). Another key insight from this study was the addition of FGF8b during endoderm anteriorization at day 4.5 which results in a 5-fold increase of *FOXN1* expression. Remarkably, TECs derived from hESc following this protocol could be maintained for up to 30 days in classical 2D culture and they expressed the thymic markers *FOXN1*, *PAX9*, *EYA1*, *SIX1* and *AIRE* at similar levels than in the human fetal thymus. Another crucial aim is the characterization of the induced TECs at the single-cell level. After a directed differentiation protocol yielding 46% of TEPs (EPCAM+ CD205+) at day 14, Ramos et al. reaggregated the cells and perform their engraftment in nude mice. After 14 to 19 weeks, the thymic grafts were analyzed by bulk and scRNA-seq. Confirmation of further maturation in TECs was provided by high levels of expression of *HLA*-*DRA* and *DLL4*. Remarkably, the scRNA-seq data of these grafts showed a common clustering of TECs derived from iPSc with primary TECs from postnatal thymuses. However, subclustering of the TEC population revealed a distinct separation between the two types of samples, with induced TECs mainly composed of TEPs and differentiating TECs, while cells from the more mature cTEC and mTEChi clusters were originating from the primary samples.

These data shed a new light on the mechanisms leading to TEP differentiation, notably in pointing out the roles of Activin A and the Notch pathway detected by the expression of *INHBA* and *DLK1* in the TEP cluster. This direct differentiation protocol allows precise differentiation of iPSc into TEPs that mature into functional TECs *in vivo* with a transcriptomic profile close to primary TECs. Further application of scRNA-seq techniques to TEC differentiation could lead to a deeper understanding of the mechanisms driving the generation of the diverse TEC populations and leverage this new knowledge to differentiate specific TEC subpopulations.

Together, TEC differentiation from pluripotent stem cells has shown significant improvement in recent years with a continuous improvement of the protocols giving rise to TEPs, with greater yield and purity. Conversely, less progress was achieved in the approaches aiming to obtain mature TECs from TEPs. Regarding this stage of maturation, important clues came from the study of the thymic crosstalk with the finding that the interactions between TECs and developing thymocytes are necessary to the maturation of TECs. It was also shown that the thymic crosstalk could be mimicked *in vitro* using cytokines including but not restricted to RANKL. At a functional level, these induced TECs (iTEC) can support the maturation of thymocytes and reconstitute the T cell compartment *in vivo*. T cells cocultured with iTEC proliferate and secrete cytokines after stimulation. They are also able to improve skin graft rejection, thus showing evidence of functionality. Interestingly, nTreg can also be generated by iTEC grafts and induce immune tolerance to syngeneic skin grafts.

Although all the above protocols succeeded in generating TEPs with various efficiency, considerable obstacles still need to be addressed to obtain a functional thymic organoid *in vitro*. This highlights the fact that our current level of control over thymic iPSc differentiation is still incomplete and that most of the modulated pathways in these protocols may not be necessary nor sufficient.

Heterogeneity of the directed differentiation products is also a crucial stake for clinical applications since undifferentiated iPSc can lead to teratoma formation after transplantation. Purification strategies and treatment by selective anti-iPSc molecules such as YM155 (133) are promising approaches to mitigate this risk. This differentiation heterogeneity could also counter-intuitively positively affect the generation of iPSc-derived TECs. As described above, multiple cell types collaborate for thymic functionality. Thus, a differentiation strategy yielding not only isolated TECs but also thymic fibroblast or even T progenitors and dendritic cells would improve TEC differentiation.

Finally, a long-term culture system accurately reproducing *in vivo* thymic microenvironment still needs to be developed. Although TECs derived from iPSc can be maintained in classical 2D culture for up to 30 days (133), a culture system closer to the 3D sponge-like structure of the thymic stroma could significantly improve TEC differentiation and viability.

## 3D Culture and Organoids

In classical 2D culture, primary TECs show a progressive loss of *AIRE* and *FOXN1* expression (134) and of their ability to express the full set of TRA genes (135). Since the 3D structure is an important factor for TEC maintenance (136), recapitulating such an organization in culture systems for thymic differentiation could improve yields and viability of iTECs. Several 3D models have been developed and tested on primary TECs. One of them relies on the coculture of mTECs with dermal fibroblasts embedded in a fibrin hydrogel, allowing proliferation, phenotype conservation, and further maturation of these cells (135). In comparison to simpler 2D culture systems, this organotypic 3D culture allows mTECs to keep their primary ability to express TRA genes. Other synthetic hydrogels have been developed to reconstitute the thymic microenvironment, such as a self-assembling synthetic hydrogel formed by EAK16-II/EAKII-H6 peptides (137). This gel was shown to induce the organization of primary TECs in 3D clusters and maintain the expression of *FOXN1* and *EPCAM*. After transplantation in nude mice, these hydrogel-embedded TECs supported the generation of T cell populations that were able to induce self-tolerance, as assessed by mixed leukocyte reactions. However, no cortico-medullary TEC segregation was observed in the reconstituted thymus, and the generated T cells were considerably biased toward CD8+ SP T cells. Additional systems were also generated to support the 3D culture of TECs, such as those based on fibronectin functionalized fibrous meshes (138), gelatin spheric microgels (139) or type I collagen hydrogel (140). Despite the various protein compositions of their matrices, these systems supported an enhanced proliferation, spreading and maintenance of the main TEC populations. However, the collagen hydrogel seeded with mice TEC did not seem to support thymopoiesis *in vivo* (140). Although synthetic hydrogels are a promising tool to culture TECs, more thorough research and optimization are needed to accurately mimic the thymic microenvironment.

An alternative approach to these synthetic systems is to directly use the primary thymus matrix to benefit from the native diversity of its constituting proteins and 3D structure. Decellularization of primary thymic lobes results in scaffolds that conserve the microstructure and the protein composition of the thymic microenvironment (141–143). These scaffolds support TEC growth and differentiation with the conservation of FOXN1 expression and the reformation of distinct medullary and cortical compartments. Moreover, after engraftment, the reconstituted thymuses can support the generation of functional T cells that can induce donor-specific tolerance in a skin graft model (141, 143). However, these approaches are dependent on a primary source of mouse or human thymuses, thus hindering clinical use.

Lastly, beside TEC differentiation, recent studies aiming to obtain functional T cells from hematopoietic progenitors or iPSc have also highlighted the importance of 3D culture systems to leverage obstacles in order to generate TEC organoids. Development of artificial thymic organoids (ATO) (144–146) has been achieved through reaggregation of a bone marrow stromal cell line engineered to express the Notch ligand Dll4 with either hematopoietic stem cells or mesodermal progenitors derived from iPSc. The cocultured cells formed a 3D structure cultured in an air-liquid interface. Using bone marrow stem cells from mice of three different backgrounds, the ATO system was able to consistently reproduce thymopoiesis, and generate mature single-positive CD4 and CD8 cells (145, 146). Remarkably, this system resulted in comparable results when using mesodermal progenitors derived from hESc and human iPSc (145). Thus, thymic organoids can recapitulate T cell differentiation *in vitro*, therefore showing the importance of 3D structure in comparison to less efficient 2D cocultures (147). However, CD4 single-positive T cell frequency was lower than expected, probably because of the absence of TECs in this system, resulting in an impaired MHCII signaling. Another crucial drawback is the conservation of numerous clones naturally eliminated in the thymus, illustrating the absence of negative selection in the ATOs.

## Clinical Applications to APECED and Perspectives

**Figure 4:**
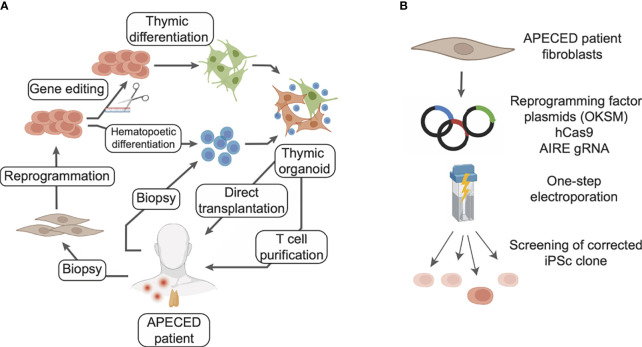
Potential cellular therapy strategies for treating APECED with induced TECs to restore thymic functionality. **(A)** Fibroblasts from the patient can be reprogrammed to iPSc and the defective gene corrected with gene editing tools. Differentiation of these iPSc into thymic epithelial cells, in combination with T cell progenitors differentiated from the same iPSc or directly purified from patient blood, would allow generation of artificial thymic organoids. These organoids could be either directly transplanted or used to generate competent T cells *ex vivo* to reconstitute the patient immune system. In this scenario T cell progenitors could be purified from patient blood or differentiated from patient iPSc. **(B)** Gene editing of a deficient gene from a patient can rely on CRISPR/Cas9 and be combined with reprogramming steps for the obtention of iPSc.

Congenital pathologies affecting TEC development and function lead to severe conditions of autoimmunity or lymphopenia. APECED is caused by loss-of-function mutations in the *AIRE* gene resulting in a general autoimmune syndrome. Patients receive a personalized combination of treatments targeting the symptoms and leading to clinical improvements. However, no curative strategy is available yet, and APECED patients still risk premature death. Remarkably, the recent breakthroughs in TEC generation from iPSc lead to new perspectives for treatment of APECED (**Figure 4A**). Reprogramming patient cells and correcting the *AIRE* mutations through gene editing techniques would produce iPSc that could be used to regenerate 3D thymic tissues. Transplantation of these autologous engineered thymic tissues would restore thymic function and limit the risk of autoimmunity. Indeed, the transplanted tissues would be syngeneic and T cells generated would be educated to the patient’s autoantigen repertoire, reconstituting its immune system (**Figure 4A**).

Reprogramming of somatic cells from APECED patients has been shown feasible despite the role that AIRE plays in the regulation of ESc pluripotency and in their self-renewal (148, 149). Indeed iPSc have recently been generated from APECED patient cells (150), showing that the *AIRE* R257X mutation does not impair cell reprogramming, iPSc proliferation and pluripotency. In this study, PBMCs from 2 female APECED patients were transduced by a Sendai virus vector, yielding 10 iPSc clones in which the *AIRE* mutation was confirmed. These iPSc show similar proliferation and expression of pluripotency markers than iPSc with functional *AIRE*, therefore validating the use of iPSc-based approaches in APECED. If *AIRE* has not been reportedly corrected in iPSc, this approach has been demonstrated in several models of monogenic pathologies, affecting diverse organs such as retina, kidney and liver (151–153). In these studies, the mutated genes *RPGR*, *IFT140* and *LDLR* causing Retinitis pigmentosa, nephronophthisis and homozygous familial hypercholesterolemia, respectively, were corrected in iPSc from patient cells. The corrected iPSc were then differentiated into retinal organoids, kidney organoids or hepatocyte-like cells, all of them showing a rescued phenotype and functionality (151–153). The CRISPR/Cas9 system was used for gene edition in these 3 studies, delivering plasmids and Cas9 to the iPSc by electroporation (152, 153), or in a one-step protocol during reprogramming (151). In the latter, patient dermal fibroblasts are electroporated by two pulses at 1,400 V for 20 ms with a cocktail of plasmids coding for the reprogramming factors, a guide RNA for the target gene, its corrected sequence, and a spCas9-gem. This specific Cas9 variants have been developed for gene editing iPSc (154). Thus, given those converging clues, using CRISPR/Cas9 to edit *AIRE* in iPSc derived from APECED patients could be a valid curative strategy (**Figure 4B**).

Beyond APECED, other pathologies affecting the thymus are also caused by genetic defects. DiGeorges syndrome is caused by a microdeletion of the TBX1-containing chromosome region 22q11.2, nude SCID by mutations in the FOXN1 gene and Otofaciocervical Syndrome type 2 in the PAX1 gene (155). These conditions lead to partial or total athymia and life-threatening lymphopenia. Curative treatments of these diseases could also rely on transplantation of autologous engineered thymic tissues to restore thymic function while limiting the risk of autoimmunity. Nonetheless, a crucial limitation is the long lapse of time needed to reprogram iPSc and to differentiate them into functional thymic tissue. Applied to APECED, early diagnosis would be crucial, since this approach cannot cure the damage already caused by autoimmunity, even though promising immunotherapies are emerging to treat autoimmune manifestations (156). For pathologies causing lymphopenia, early diagnosis would also be vital to limit the risk of potentially lethal infections during the time needed to generate the engineered thymic tissues. To envision the use of such therapies, additional challenges would need to be met with the need to develop clinical-grade differentiation protocols not relying on any xenogenous reactives and based on well-accepted synthetic hydrogels for 3D culture. Finally, the risk of transplanting iSPc-derived TECs should be carefully assessed to limit teratoma formation, with purification of differentiated cells and anti-pluripotency treatment.

Because of the complex interactions between cell populations in the thymus, the crucial importance of its 3D organization and the still-improving understanding of TEC biology, generation of *in vitro* culture systems closely reproducing the thymus is a major challenge. In recent years, great advances have been made in the understanding of thymus organogenesis, the generation of TECs from pluripotent stem cells and 3D culture systems. These complementary progress will very likely result in preclinical applications for the treatment of pathologies affecting T cell development in the thymus.

## Author Contributions

NP and MG designed/outlined the manuscript; NP wrote the manuscript and MG edited the manuscript.

## Funding

NP was supported by “la fondation d’entreprise ProGreffe” and by the EJP-Rare Disease JTC2019 program TARID project (ANR-19-RAR4-0011-05) to MG.

## Conflict of Interest

The authors declare that the research was conducted in the absence of any commercial or financial relationships that could be construed as a potential conflict of interest.

## Publisher’s Note

All claims expressed in this article are solely those of the authors and do not necessarily represent those of their affiliated organizations, or those of the publisher, the editors and the reviewers. Any product that may be evaluated in this article, or claim that may be made by its manufacturer, is not guaranteed or endorsed by the publisher.

## Abbreviations

AIRE, autoimmune regulator; APECED, polyendocrinopathy candidiasis ectodermal dystrophy syndrome; ATO, artificial thymic organoid; DN, double negative CD4-CD8- thymocyte; DP, double positive CD4+CD8+ thymocyte; ESc, embryonic stem cells; ETP, early thymic progenitor; iPSc, induced pluripotent stem cells; mTEChi, MHC-II high medullary thymic epithelial cell; nTreg, natural regulatory T cell; RA, retinoic acid; RTE, recent thymic emigrant; SCID, severe combined immunodeficiency; TCR, T cell receptor; TEC, thymic epithelial cells; TEP, thymic epithelial progenitor; TRA, tissue restricted autoantigen; 3PP, third pharyngeal pouch.
